# Melatonin-enabled omics: understanding plant responses to single and combined abiotic stresses for climate-smart agriculture

**DOI:** 10.1080/21645698.2026.2614130

**Published:** 2026-01-27

**Authors:** Ali Raza, Yiran Li, Sidra Charagh, Chunli Guo, Mengkai Zhao, Zhangli Hu

**Affiliations:** a Guangdong Key Laboratory of Plant Epigenetics, College of Life Sciences and Oceanography, Shenzhen University, Shenzhen, China; b College of Physics and Optoelectronic Engineering, Shenzhen University, Shenzhen, China; c Shenzhen Engineering Laboratory for Marine Algal Biotechnology, Guangdong Technology Research Center for Marine Algal Biotechnology, Longhua Innovation Institute for Biotechnology, College of Life Sciences and Oceanography, Shenzhen University, Shenzhen, China; d Guangdong Provincial Key Laboratory of Applied Botany, Key Laboratory of National Forestry and Grassland Administration on Plant Conservation and Utilization in Southern China, South China National Botanical Garden, State Key Laboratory of Plant Diversity and Specialty Crops, South China Botanical Garden, Chinese Academy of Sciences, Guangzhou, China; e Guangdong Provincial Key Laboratory of Functional Substances in Medicinal Resources and Healthcare Products, School of Life Sciences and Food Engineering, Hanshan Normal University, Chaozhou, China

**Keywords:** Biostimulant, climate change, genetic engineering, multi-omics, phytomelatonin, stress combination

## Abstract

Climate change-driven single and combined abiotic stresses pose escalating threats to sustainable, climate-smart agriculture and global food security. Melatonin (MLT, a powerful plant biostimulant) has established noteworthy potential in improving stress tolerance by regulating diverse physiological, biochemical, and molecular responses. Therefore, this review delivers a comprehensive synopsis of MLT-enabled omics responses across genomics, transcriptomics, proteomics, metabolomics, miRNAomics, epigenomics, phenomics, ionomics, and microbiomics levels that collectively regulate plant adaptation to multiple abiotic stresses. We also highlight the crosstalk between these omics layers and the power of integrated multi-omics (panomics) approaches to harness the complex regulatory networks underlying MLT-enabled stress tolerance. Lastly, we argue for translating these omics insights into actionable strategies through advanced genetic engineering and synthetic biology platforms to develop MLT-enabled, stress-smart crop plants.

## GRAPHICAL ABSTRACT

**Figure:**
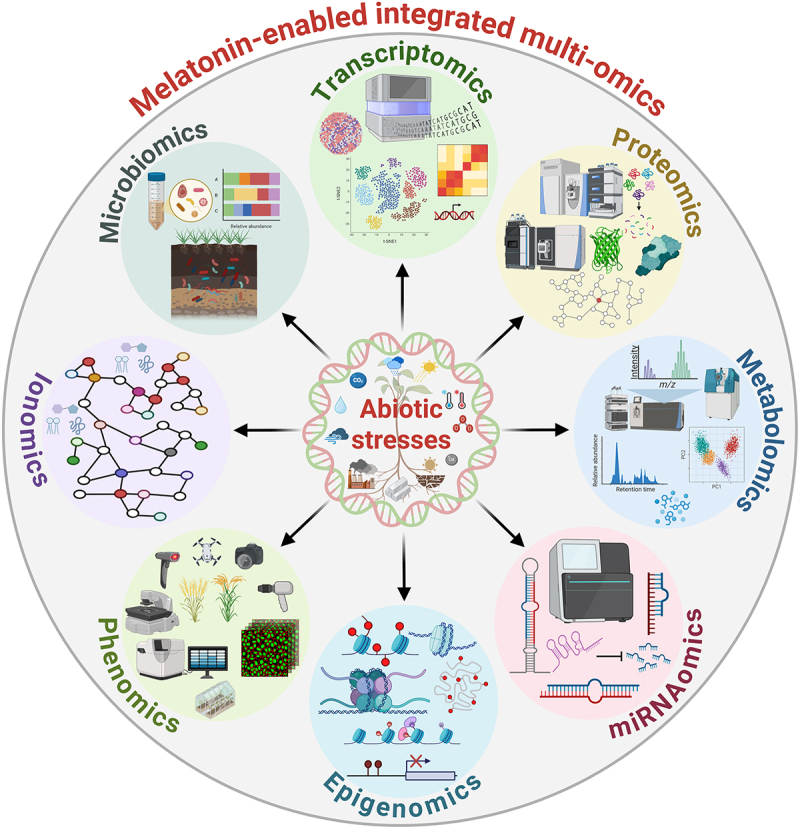

## 1. Introduction

Climate change is exerting massive pressure on global agriculture by intensifying the occurrence, duration, and overlap of abiotic stresses, e.g., drought, flooding, salinity, temperature extremes, elevated carbon dioxide, organic pollutants, metal toxicity, nutrient imbalance, and others. These stresses severely restrict crop growth, development, and productivity, posing a major challenge to food and nutritional security.^1,2^ Notably, plants in natural environments frequently face various stresses simultaneously, i.e., multifactorial stress combination (*n* ≥2), rather than separately, which results in compound physio-biochemical and molecular trade-offs that further reduce yield.^1,3–5^ Therefore, designing and breeding stress-smart crop varieties is not only a scientific urgency but also a stipulation for sustainable agricultural production in the face of unpredictable changing climates (see Section 2 for detailed arguments on climate-driven stress challenges).

**Figure 1.**
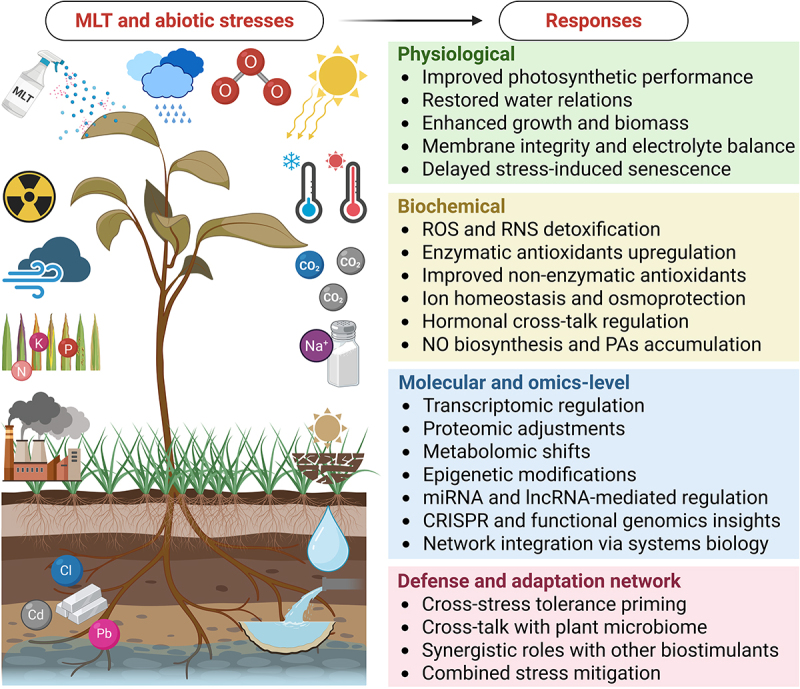
Melatonin (MLT)-enabled plant responses to diverse abiotic stresses at different levels. Created with BioRender.com.

In recent years, melatonin (“MLT,” *N*-acetyl-*5*-methoxytryptamine) has developed as a powerful and versatile “biostimulant” or signaling molecule, that is capable of boosting tolerance against diverse abiotic stresses.^6–10^ Previous evidence from physiolo-biochemical and molecular investigations has confirmed that MLT application advances photosynthesis, maintains redox homeostasis, regulates ion balance, modulates phytohormone signaling, and activates antioxidative defense systems under multiple abiotic stress conditions (Figure 1), as reviewed elsewhere.^6–17^ Mechanistically, MLT works through a multidimensional signaling cascade that integrates early redox sensing with downstream phytohormonal and transcriptional reprogramming in major crops. In economically prominent plant species, such as rice^18,19^, wheat^20,21^, and tomato^22–24^, MLT is reported to trigger rapid calcium and nitric oxide signaling, activate mitogen-activated protein kinase (MAPK) pathways, and function as both a direct reactive oxygen species (ROS) scavengser and a redox signal amplifier. These early actions induce corresponding upregulation of antioxidant enzymes (e.g., SOD, CAT, APX, and GR), stabilization of photosystem II, and protection of cellular membranes.^18–24^ Furthermore, MLT fine-tunes phytohormone networks and ion-transport systems (e.g., *SOS1*, *NHX*, *HKT1*, and aquaporins), which results in improved stomatal regulation, ionic homeostasis, and water use efficiency under stress. In brief, these interconnected mechanisms sustain carbon assimilation, preserve assimilate partitioning, and finally support yield stability under stressed conditions, as reviewed across cereal and horticultural crops.^6–19,23^ Since MLT functions as a signaling molecule, as supported by these conventional mechanisms, a rising body of literature, mainly over the past five years, signifies that MLT-treated plants exhibit diverse omics-driven responses across genomic, transcriptomic, proteomic, metabolomic, epigenomic, microbiomics, phenotypic, or ionomics levels that help discover the new mechanistic insights into stress adaptation and acclimation (see Section 3 for omics-driven responses to MLT treatments).

Multi-omics (also called “panomics”) tools are considered a modern way forward in “plant stress biology,” which enables the high-resolution, systems biology-level examination of genes, proteins, metabolites, and other regulatory elements that govern stress responses. In recent years, these tools have discovered unique networks and novel molecular elements underlying tolerance to both single and combined abiotic stresses.^5,25–32^ Though omics tools have been extensively exploited to study stress responses in plants; nevertheless, their application in understanding MLT-mediated tolerance mechanisms across different plant species remains scattered and less explored. Therefore, this review comprehensively examines recent advances in MLT-enabled omics responses and understands how these insights contribute to the development of climate-smart, future crop plants.

We assume that MLT-enabled omics responses provide critical molecular signatures and regulatory elements that can be harnessed to enhance stress tolerance in plants. Considering the power of omics tools, this review (1) synthesizes current findings on how MLT modulates plant responses to abiotic stress across omics layers, (2) highlights major omics-driven insights from MLT-treated plants, and (3) discusses future directions for integrating MLT-enabled stress biology with omics and biotechnological tools to engineer stress-smart crops for sustainable agriculture and safeguard future food security.

## 2. Climate Change, Single and Combined Abiotic Stresses: Escalating Threats to Sustainable Agriculture and Global Food Security

Unpredictable climate change is one of the most critical challenges threatening global food and nutritional security, with severe consequences for sustainable agriculture, especially in developing countries.^2,3^ Among different factors, extreme temperatures, erratic rainfall, floods, droughts, soil salinization, and elevated carbon dioxide levels gradually upset crop productivity, quality, and tolerance potential.^2,3,33^ These stresses, whether occurring independently or in combination, impair key processes in plants^1–4^, which makes it challenging to meet the projected 60% increase in food demand by 2050 (https://www.iaea.org/topics/food-security-and-climate-change).

Notably, agriculture endures the livelihoods of over 2.5 billion people globally. Yet, recent estimates demonstrate that 713–757 million peoples faced malnutrition in 2023, along with ~2.4 billion facing food insecurity, and this alarming trend expected to deteriorate with future climate unpredictability (see FAO reports for more insights: https://www.fao.org/interactive/state-of-food-security-nutrition/en/.; and https://www.fao.org/newsroom/detail/world-must-look-to-south-america-success-in-reducing-hunger-fao-chief-economist/en). For instance, elevated carbon dioxide can stimulate growth in some C_3_ crops, e.g., rice (*Oryza sativa* L.) and wheat (*Triticum aestivum* L.), and its effects are complex and often offset by complementary heat, drought, and nutrient limitations.^34–36^

Single stress factors, such as drought^37–40^, salinity^32,41,42^, or extreme temperatures^15,30,43–45^, are already imposing yield penalties. However, combined abiotic stress conditions, e.g., drought + heat, drought + salinity + heat, salinity + nutrient imbalance, etc., result in even massive threats, which often cause natural or synergistically negative effects on plant physiology and productivity.^1,3–5,31,46^ Under combined stresses, plants display contradictory physiological responses, e.g., stomata may close in response to drought but stay open under heat, which leads to inhibited water use and higher tissue temperatures.^46–48^ These interactions can lead to increased oxidative stress, disrupted energy balance, impaired photosynthesis, and severe reproductive failure.

Moreover, under combined abiotic stresses, the stress response often differs between vegetative and reproductive tissues, with the latter being more sensitive and crucial for yield. Recent studies suggest that plants adopt tissue-specific mechanisms, such as “differential transpiration,” to mitigate damage in reproductive organs while reducing stress “trade-offs” in leaves.^47,49^ This phenomenon highlights the need for targeted research to harness stress-specific and tissue-specific responses, which could form the basis for designing climate-smart crops.

To sustain agricultural productivity in the face of these escalating threats, there is an urgent need to develop climate-smart crops and cropping systems that can withstand multiple abiotic stresses. This includes leveraging integrated strategies from stress physiology to omics technologies, to improve crop adaptation and maintain yield stability under a changing climate.^2,5^ In summary, climate change is no longer a distant risk but a current reality that is reshaping agricultural production. Thus, addressing the complexity of single and combined abiotic stresses is essential to secure the future of sustainable agriculture and global food systems.

## 3. Melatonin-Enabled Omics Responses Across Plant Species: Toward Understanding Stress Adaptation and Tolerance Mechanisms

**Figure 2.**
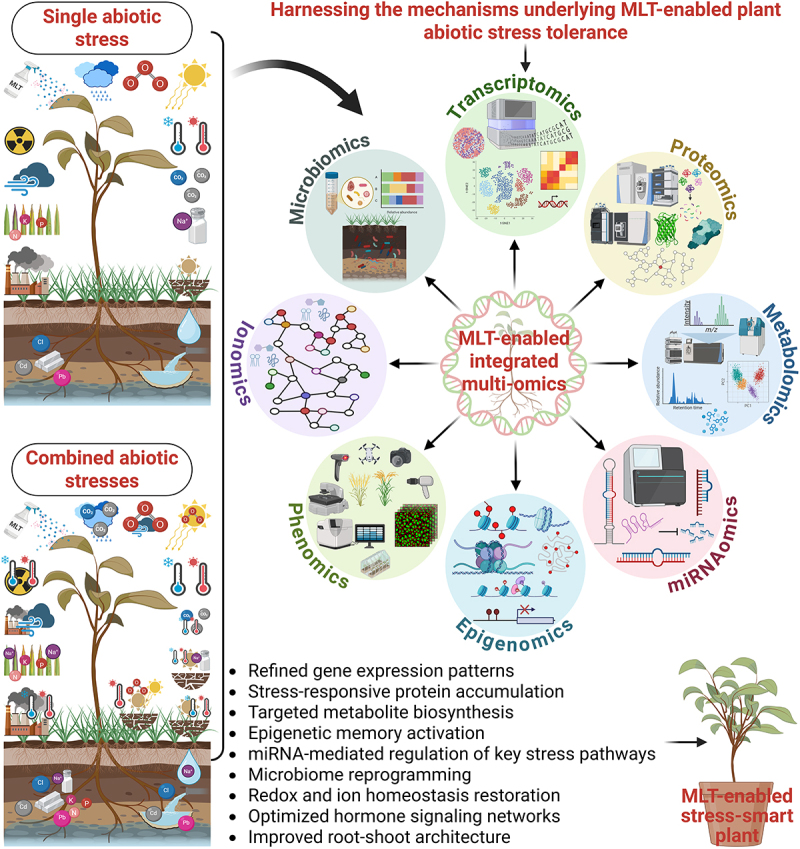
A graphic scheme shows how melatonin (MLT)-enabled multi-omics tools can be employed to harness and reprogram the mechanisms by which plants respond and tolerate single or combined abiotic stresses. MLT application activates a range of omics layers that mutually shape gene expression, metabolite accumulation, protein abundance, adaptive phenotypes, and others. These harmonized mechanisms ultimately lead to the development of MLT-enabled, stress-smart plants. Created with BioRender.com.

**Figure 3.**
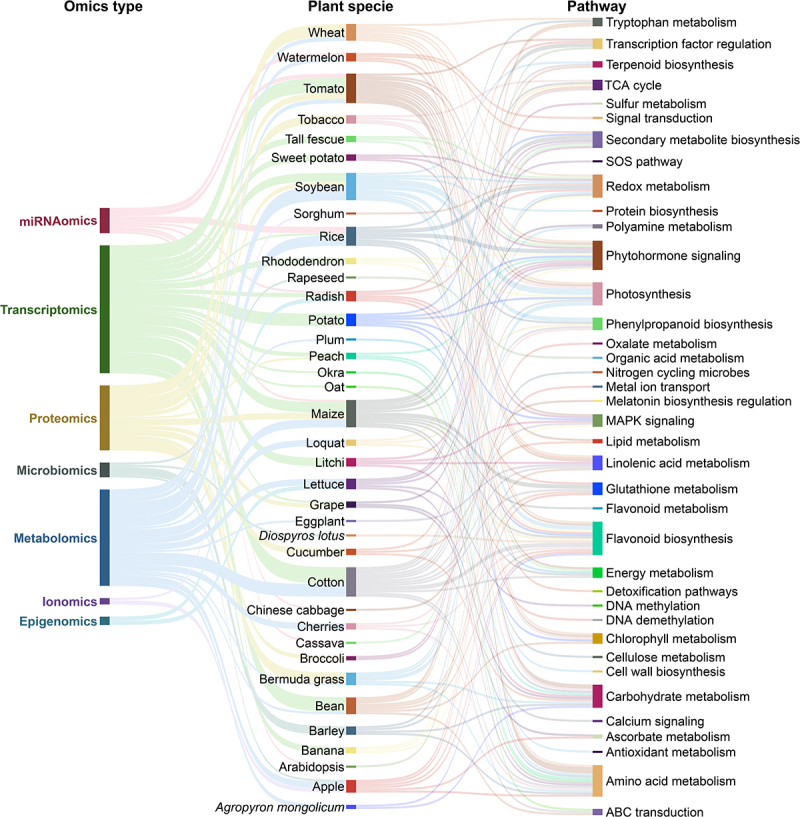
A comprehensive Sankey diagram illustrates the connection between melatonin-enabled omics techniques, plant species, and stress-responsive pathways under single abiotic stress conditions. This plot highlights both unique and shared molecular pathways modulated by melatonin. Data was obtained from all the studies cited within the main text body of Sections 3.1 to 3.9 and Table 1.

With the help of recent advances in multi-omics tools, we can now decipher the complex and profound effects of MLT on plant systems, i.e., transcriptional reprogramming, proteomic shifts, metabolic rewiring, ionomic and epigenetic adjustment, and microbiome modulation. In this context, Figure 2 highlights a conceptual model of how different MLT-enabled omics layers rewrite an integrated defense network to reprogram plant stress responses. To further map the landscape of MLT-enabled changes, a Sankey diagram spotlights the unique and overlapping pathways regulated by MLT across omics types and plant species (Figure 3). In the subsequent sections, we have discussed the MLT-enabled responses at different omics levels.

**Table 1.** Insights from melatonin-enabled omics studies under different stress conditions.

Stress type	Plant specie	MLT dose	Tissue	Specific candidate genes, proteins, and metabolites (if any)	Pathways regulated by MLT	References
**Transcriptomics**						
Heat	Tall fescue (*Festuca arundinacea*)	50 mM	Leaves	*FaHSFA3*, *FaAWPM* and *FaCYTC2*	DNA, RNA and protein degradation, redox, energy, and hormone metabolisms	50
Heat	Rhododendron	200 µmol L^−1^	Leaves	*RhPGR5A*, *RhATPB*, *RhLHCB3*, and *RhRbsA*	Photosynthesis, carbon fixation, phytohormone signal transduction, and flavonoid biosynthesis	51
Heat	Potato (*Solanum tuberosum*)	50, 100, 150 and 200 µmol L^−1^	Leaves	*Lcha1*, *Lcha2*, *Lcha3*, *Lcha4*, *Lchb1*, *Lchb2*, *Lchb5*, *POR1*, *CHLM*, *CHLG*, *CHLD*, *APXT*, *SODCP.2*, *GRF3*, *GRIP*, *CAT4*, *SODB*, *LAX5*, *SAUR*, *ARG7*, *NTF4*, W*RKY22*, *RBHD*, and *RBHE*	Phytohormone signal transduction, MAPK signal pathway, biosynthesis of secondary metabolites and phenylpropane compounds, endoplasmic reticulum protein processing, photosynthesis-antenna protein, and porphyrin and chlorophyll metabolism	52
Heat	Sweet potato (*Ipomoea batatas*)	100 μM	Leaves	*IbWRKY33*, *IbWRKY7*, *IbWRKY40*, *IbHSP21*, *IbHSA32*, and *IbMPK3*	Redox regulation and nicotinate and nicotinamide metabolisms, and MAPK and phytohormone signal transduction pathways	53
Cold	Grape (*Vitis vinifera*)	150 μM	Seedlings	*VIT_00s0179g00150*, *VIT_18s0041g02080*, *VIT_16s0050g00980*, *VIT_07s0031g02480*, *glutathione S-transferase*, *UDP-glycosyltransferase*, *calcium-binding protein*, *cytochrome P450*, *phytohormone*, and *ABC transporter*	Metabolic process, protein modification, chloroplast complex, carbohydrate transport, and oxidoreductase activity pathways	54
Cold	Litchi (*Litchi chinensis*)	100 μM	Leaves	*LITCHI002002*, *LITCHI010152*, *LITCHI014934*, *LITCHI010388*, *LITCHI006790*, *LITCHI010992*, *LITCHI010993*, *LITCHI024087*, *LITCHI003095*, *LITCHI024086*, and *LITCHI024085*	Starch and sucrose, phytohormone (auxin, ABA), MAPK, and alpha-linolenic acid metabolism pathways	55
Cold	Tomato (*Solanum lycopersicum*)	100 μM	Leaves	*FEZ*, *ATHB-52*, *BZIP34*, *ZAT9*, *WRKY49*, *HSFA2B*, and *DOF5.3*	Carbohydrate, terpenoid polyketone, amino acid and lipid metabolisms, genetic information processing, and signal transduction pathways	56
Cold	Bananas (*Musa acuminata*)	500 μmol L^−1^	Fruits	*MaRBOH*, *MaSOD*, *MaCAT*, *MaAPX*, *MaGPX*, and *MaGR*	Redox metabolism, secondary metabolites biosynthesis, and MAPK and phytohormone signaling pathways	57
Sodic Alkaline	Tomato (*Solanum lycopersicum*)	0.5 μM	Roots	*DREB1α* and *IAA3*	Phytohormone signaling pathways	58
Alkaline	Soybean (*Glycine max*)	100 μmol L^−1^	Vegetative growth and pod filling stage	Different TFs (P2/ERF, bHLH, MYB, WRKY, NAC, and C2H2), and genes such as *SoyZH13_08G026600*, *SoyZH13_13G093900*, *SoyZH13_10G164700*, *SoyZH13_10G164800*, *SoyZH13_19G230400*, and *SoyZH13_07G055700*	Ribosome biogenesis, photosynthesis, peroxisome, and phytohormone signaling pathways, redox metabolism, phenylpropanoid and flavonoid biosynthesis	59
Salinity	Okra (*Abelmoschus esculentus*)	50 mM	Leaves, roots, and stems	Different TFs (MYB, WRKY, NAC, etc.)	Nitrogen, sulfur, alanine, aspartate, and glutamate metabolisms	60
Salinity	Cotton (*Gossypium hirsutum*)	50, 100, 200, and 500 μM	Leaves	*hemF*, *hemH*, *hemL*, *hemC*, *ChlI*, *LHCB4*, *LHCB3*, *LHCA2*, *petJ*, *petF*, *petH*, *PsbP*, *PsbQ*, *PsbR*, *AUX1*, *SUAR*, *PLA2G*, and *OPCL5*	Linolenic acid metabolism, flavonoid and phenylpropanoid biosynthesis, and photosynthesis-related and phytohormone signaling pathways	61
Salinity	Rice (*Oryza sativa*)	10 μM	Leaves	Different TFs (AP2/EREBP, HB, and WRKY), and *OsEXPB2*, *Hsp40*, *OsBBX20*, and *OsLTP2.12*	Transcriptional cascade and phytohormone signaling pathways	62
Salinity	Common bean (*Phaseolus vulgaris*)	100 µmol L^−1^	Leaves	*Phvul.002G074300*, *Phvul.006G197900*, *Phvul.001G187800*, *Phvul.001G218000*, *Phvul.006G197900*, *Phvul.005G111700*, *Phvul.007G228200*, *Phvul.007G228200*, etc.	Flavonoid, terpenoid, porphyrin, tryptophan, and chlorophyll, glutathione, linolenic acid metabolisms, zeatin biosynthesis, and ABC transduction	63
Drought	Maize (*Zea mays*)	100 μM	Leaves	Different TFs (WRKY, AP2/ERF-ERF, MYB, NAC, and bZIP)	Glutathione metabolism, calcium signaling transduction, and jasmonic acid biosynthesis	64
Drought	Naked oat (*Avena nuda*)	100 µM	Leaves	*PYL*, *PP2C*, *ABF*, *SNRK2*, and *IAA*	ABA signal transduction pathway	65
Drought	Tomato (*Solanum lycopersicum*)	50 µM	Leaves	Different TFs (MYB, bHLH, NAC, MADS, WRKY, AP2, C2H2, and bZIP), and genes (*TGL4*, *CYP1A2*, and *LOX2S*)	Phytohormone signal transduction, photosynthesis-antenna proteins, and linoleic acid metabolism	66
Drought	Maize (*Zea mays*)	100 μM	Roots	Different TFs (ERFs, NACs, MYBs, and bHLHs), and genes including (PAL, C4H, 4CL, HCT, CHS, CHI, F3′5′H, DFR, ERF4, ERF81, and ERF110)	Flavonoid biosynthesis and phytohormone signal transduction pathways	67
Waterlogging	Peach (*Prunus persica*)	200 μM	Roots	Different DNA-binding TFs (AP2/ERF, HSF, and WRKY)	Glycolysis/gluconeogenesis pathway, and metabolism of amino sugar and nucleotide sugar, fructose and mannose, alanine, aspartate and glutamate, and phytohormone signal transduction	68
Cadmium	Radish (*Raphanus sativus*)	10, 25, 50, 100, and 200 μmol L^−1^	Roots	*YSL*, *HMA*, *ABC* transporters, and *MT1*	Glucosinolate and flavonoid biosynthesis, cysteine, methionine, and glutathione metabolisms	69
Cadmium	Cotton (*Gossypium hirsutum*)	15 µM	Leaves	Different TFs (AP2-EREBP, NAC, bHLH, WRKY, and Trihelix), and genes such as *ripA* and *IDH1*	Chloroplasts and mitochondria regulated carbohydrate and energy metabolism, and cellular components and biological processes	70
Copper	Tomato (*Solanum lycopersicum*)	100 μM	Leaves	Genes associated with membrane and cell part, binding, catalytic, and antioxidant activities	Photosynthesis antenna proteins, phytohormone signal transduction, plant MAPK signaling pathways, and biosynthesis of various secondary metabolisms	71
**Proteomics**						
Drought	Maize (*Zea mays*)	0, 1, 10, 100, and 1000 μM	Leaves	Different genes associated with photosynthesis, carbon fixation, biosynthesis of secondary metabolites and amino acids	Glutathione, carbon fixation and carbon metabolisms, biosynthesis of secondary metabolites, and TCA cycle	72
Heat	Tobacco (*Nicotiana tabacum*)	50 μM	Leaves	HemA, HemB, HemD, HemY, ChlE, ChlH, ChlM, DVR, POR, PsbE, PsbH, PsbO, PsbE, PsbH, and PsbR, PsbO, PsbQ, PsbP, PsaA, PsaB and others	Chlorophyll biosynthetic pathway, PSII photochemical reaction, TCA cycle, and carbohydrate metabolism	73
Oxidative	Bermuda grass (*Cynodon dactylon*)	NA	Seedlings	Different proteins involved in redox metabolism	Polyamine, carbohydrate, photosynthesis, redox, and amino acid metabolisms, and ribosome pathway	74
Oxidative	Grape (*Vitis vinifera*)	50, 100, 200, and 400 μmol L^−1^	Skin	VvADC, VvODC, VvNAC, VvSPDS and VvCuAO	Arginine and proline, ascorbate and aldarate, lysine degradation pathways, and polyamine metabolisms	75
Drought	Wheat (*Triticum aestivum*)	100 μM	Shoots	LOX1.5, LOX2.1, HY5, MYB86, 4CL2, P5CS1, CCR2, PME53 and SUS4	Oxidative phosphorylation, ribosome, carbon fixation in photosynthetic organisms, photosynthesis, carbon, porphyrin and chlorophyll, and linoleic acid metabolisms, JA biosynthesis	76
**Metabolomics**						
Cold	Bermudagrass (*Cynodon dactylon*)	100 μM	Leaves	Sugars (arabinose, mannose, glucopyranose, maltose, and turanose) and organic acid (propanoic acid)	Photosynthesis and metabolism-related pathways	77
Cold	Plum (*Prunus salicina* Lindl.)	1 mmol L^−1^	Fruit	Phenolics, anthocyanins, carotenoids, and phenylpropanoids	Energy metabolism	78
Salinity	Rice (*Oryza sativa*)	0.5, 1, 5, 10, 50 and 100 µM	Seeds	Organic acids and amino acids	Carbohydrate and organic acid metabolisms	79
Salinity	Cotton (*Gossypium hirsutum*)	10 μmol L^−1^	Leaves	Flavonoids, sugars, alcohols, indoles, alkaloids, melatonin, organic acids, lipids, and terpenoids	Terpenoid material synthesis, the phosphatidyl inositol signaling system, and metabolisms of alanine, tyrosine, biotin, inositol phosphate, arginine and proline, and biosynthesis of flavonoid synthesis, the TCA cycle, pantothenic acid and CoA, arginine synthesis, alanine, glutamic acid and aspartic	80
Salinity	Maize (*Zea mays*)	10 μM	Seeds	Secondary metabolites, nucleotides, cofactors, and vitamins	Amino acid metabolism and secondary metabolite biosynthetic pathways	81
Salinity	Common bean (*Phaseolus vulgaris*)	100 µmol L^−1^	Leaves	C05642 (N-acetyl-N-2-formyl-5-methoxycanurine), C05643 (6-hydroxymelatonin), and C05660 (5-methoxyindoleacetic acid)	Tryptophan metabolism	63
Salinity	Eggplant (*Solanum melongena*)	200 μmol L^−1^	Leaves	α-Linolenic acid, (9 R,13 R)-12-oxophytodienoic acid, 9(S)-HpOTrE, and (+)-7-iso-Jasmonic acid	α-linolenic acid metabolism pathway	82
Salinity	Lettuce (*Lactuca sativa*)	150 μM	Leaves	Flavonols, amino acids, nitrogen and sulfur compounds	Secondary metabolism, phytohormones, fatty acids and amino acids biosynthesis	83
Drought	Soybean (*Glycine max*)	100 µmol L^−1^	Leaves	Secondary metabolites	Phenylpropanoid, flavonoid, isoflavonoid, and steroid biosynthesis pathways	84
Drought	Loquat (*Eriobotrya japonica* Lindl.)	150 μM	Leaves	Amino acids and organic acids	Phenylpropanoid and flavonoid metabolisms, and the TCA cycle	85
Drought	*Agropyron mongolicum*	100 mg L^−1^	Leaves	Flavonoids, phenolic acids, lipids, alkaloids, amino acids and derivatives, organic acids, nucleotides and derivatives, lignans and coumarins, terpenoids	Flavonoid biosynthesis and carbohydrate metabolism	86
Drought	Maize (*Zea mays*)	100 µM	Roots and leaves	Amino acids include proline, methionine, serine, and phenylalanine	Energy, and amino acid metabolism pathways	87
Drought	Wheat (*Triticum aestivum*)	10 mM	Leaves	Tryptamine, MT, formylanthranilate, 3-hydroxyanthranilate, 6-hydroxymelatonin, naringenin chalcone, astragalin, pinbanksin, and caffeoyl quinic acid	Tryptophan metabolism and flavonoid biosynthesis pathways	88
Copper	Melon (*Cucumis melo*)	100 μmol L^−1^	Roots	Val-Phe, l-mannomethylose, dolichyl diphosphate, uridine, raffinose, and hexadecanedioic acid	Biosynthesis of secondary metabolites, pyrimidine and linoleic acid metabolisms	89
Arsenic	Rice (*Oryza sativa*)	20 μM	Grains	Anthocyanins, flavonoids, total phenolics and ascorbic acids, and thiol-metabolites	Phenylpropanoid pathway, and phytochelatins biosynthesis	90
Cadmium	Rice (*Oryza sativa*)	100 μM	Leaves	Amino acids, citric acid, melatonin biosynthetic metabolites, and phytochromes	Biosynthesis of amino acids-related and benzoxazinoid pathways, and sphingolipid metabolism	91
Circadian	*Arabidopsis thaliana* and *Brassica nigra*	50 and 100 μM	Whole plants	16-oxo-palmitate, (2 R,3S,4S)-leucocyanidin, sugar phosphates, D-glucose 1-phosphate, and amino sugars	Pigment and polyamine biosynthesis, antioxidant, flavonoid and phenylpropanoid pathways, cytokinin signaling, sulfur metabolism, and nucleotide regulations	92
Mechanical (surface pitting)	Sweet cherries (*Prunus avium*)	400 µM	Fruits	Secondary metabolites	Biosynthesis of phenylpropanoid, stilbenoid, diarylheptanoids and gingerol, flavonoid, anthocyanin, and sphingolipid, sulfur, amino sugar, and nucleotide sugar metabolisms	93

### 3.1. Responses at Genomics Levels

Diverse genomics tools and resources have made it possible to sequence thousands of plant genomes (https://phytozome-next.jgi.doe.gov/.; https://www.ncbi.nlm.nih.gov/genome/browse#!/overview/.), which can fast-track the stress-smart genomics-assisted and fast-forward breeding^32,94^. By utilizing genomics tools, we can now identify and characterize new gene families in diverse plant species, particularly those involved in MLT biosynthesis, such as tryptophan decarboxylase (TDC), tryptophan hydroxylase (TPH), tryptamine 5-hydroxylase (T5H), N-acetylserotonin methyltransferase (ASMT), serotonin N-acetyltransferase (SNAT), and caeic acid O-methyltransferase (COMT). For instance, different *TDC*, *T5H*, *SNAT*, and *ASMT*-related genes were identified in Arabidopsis (*Arabidopsis thaliana* L.), tomato (*Solanum lycopersicum* L.), rice, and sorghum (*Sorghum bicolor* L.). Their expression was influenced under different stress conditions, i.e., light/dark, salinity, drought, and heat.^95^ A total of 37 genes, including one *MnTDC*, seven *MnT5Hs*, six *MnSNATs*, 20 *MnASMTs*, and three *MnCOMTs*, were discovered in wild mulberry (*Morus notabilis*), and provided insights into their role in MLT biosynthesis^96^. In apple (*Malus domestica* Borkh.), 37 *MdASMT* genes were discovered and upregulated under drought, heat, cold, and salinity stresses^97^. In cotton (*Gossypium hirsutum* L.), 52 *GhSNAT* genes were identified, and the MLT treatment enhances the salinity tolerance of *GhSNAT3D*-silenced plants^98^. In Chinese cabbage (*Brassica rapa*), two *BcSNAT1-2* genes responded to different phytohormones, heavy metals, salinity, and drought stresses^99^. Likewise, many other genes have been reported in various species, such as 12 *NtSNATs* genes in tobacco (*Nicotiana tabacum* L.)^100^, *SlASMTs* genes in tomato^101^, *GmCOMTs* genes in soybean (*Glycine max* L.)^102^, *CaASMTs* genes in pepper (*Capsicum annuum* L.)^103^, *CsASMTs* genes in tea (*Camellia sinensis*)^104^, *CbuCOMTs* genes in *Catalpa bungei* ^105^, *OsCOMTs* genes in rice^106^, and many more. In each study, many genes were upregulated in response to diverse abiotic stresses, and in some cases, by MLT treatment, which highlights their potential role in developing stress-tolerant future cultivars. Likewise, these genes could serve as candidates for manipulating the endogenous MLT for the proper functioning of MLT-associated pathways, mainly under stressful conditions.

In addition to MLT biosynthesis-related genes, MLT treatment also responds and upregulates the expression of other genes in plants. Here, only a few studies are highlighted as case examples. Different gene families have been reported in dragon fruit (*Selenicereus undatus* L.), including *HuMATEs* ^107^, *HuHMAs* ^108^, *HuAPXs* ^109^, *HuMADS-box*. ^110^, and *HuSBPs* ^111^ In these studies, MLT treatment increases gene expression, which ultimately enhances tolerance against metals, salinity, drought, and heat stresses. In bean (*Phaseolus vulgaris* L.), MLT application boosted the expression of different *Pvul-SABATH* genes under salinity and drought environments.^112^ In another study, *PvGPATs* genes were upregulated when subjected to combined salinity and MLT treatment^113^. In soybeans, 145 *GmB3* genes were discovered, and most of them were significantly induced by cold stress.^114^ From these studies, it can be concluded that MLT treatment plays a key role in stress tolerance by regulating the expression levels of some specific genes. Therefore, we recommend that the functional characterization of these genes could help in the design of climate-smart crop plants.

### 3.2. Responses at Transcriptomics Levels

Transcriptomics/Transcriptome is a vital approach to discover key genes and pathways correlated with traits of interest, e.g., abiotic stress tolerance. Over the last few decades, this method has been extensively employed to understand stress tolerance mechanisms at transcriptional levels across plant species, without the treatment of external chemical molecules.^115,116^ However, literature also suggests that the exogenous application of MLT significantly contributes to stress tolerance through diverse mechanisms and metabolic pathways (Table 1; Figure 3). Therefore, we discuss how MLT regulates gene expression across multiple stress-related pathways, influencing transcription factors (TFs), metabolic pathways, and signaling networks that are integral to stress tolerance (Table 1; Figure 3).

In tall fescue (*Festuca arundinacea* Schreb.), MLT (50 mM) improved heat tolerance by regulating genes associated with DNA, RNA, and protein degradation, as well as redox, energy, and hormone metabolisms^50^. This highlights the broad regulatory role of MLT in enhancing cellular stability under heat stress, which features its ability to modulate gene networks to protect plants from heat-induced damage. Likewise, in cotton, MLT (50 µM) enhanced cadmium (Cd) tolerance by activating ABC transporters, which are vital for transporting Cd ions. Furthermore, MLT modulated genes involved in photosynthesis-antenna proteins, MAPK signaling, hormone signal transductions, and various metabolic pathways, such as amino sugar and nucleotide sugar metabolisms, starch and sucrose metabolism, galactose metabolism, valine, and leucine, and isoleucine degradation.^117^ These findings shed light on how MLT contributes to enhanced metal stress tolerance by regulating transporters and metabolic networks.

In tomatoes, MLT (50 µM) activated several TFs and key pathways, including those involved in hormone signal transduction and photosynthesis, which led to enhanced drought tolerance. Also, MLT downregulated genes responsible for linoleic acid catabolism, which is linked to reducing oxidative damage under drought conditions^66^. In maize (*Zea mays* L.), MLT (100 µM) triggered the expression of TFs, such as WRKY, AP2/ERF-ERF, MYB, NAC, and bZIP, which are essential for activating drought-responsive genes. Additionally, MLT-regulated genes involved in glutathione metabolism, calcium signaling, and jasmonic acid biosynthesis, which suggest their vital role in drought tolerance^64^. The activation of these TFs and metabolic pathways suggests a complex interaction between MLT and stress signaling networks, which results in a synchronized stress response.

MLT also influences phytohormone signaling pathways to enhance stress tolerance. For example, MLT (100 µM) regulates the ABA signaling pathway, which is crucial for drought response, improving water use efficiency and stress tolerance in oat (*Avena nuda* L.)^65^. In tomato, MLT (0.5 µM) induced the expression of important genes against sodic alkaline stress, including *DREB1α* and *IAA3*, indicating its role in modulating drought and salinity tolerance through hormone-regulated pathways^58^. In addition to improving physiological indexes, MLT (50 mM) upregulated different TFs and genes involved in nitrogen, sulfur, alanine, aspartate, and glutamate metabolisms, which contributed to salinity tolerance by modulating essential metabolic pathways in okra (*Abelmoschus esculentus* L).^60^

The above-discussed studies demonstrate the multifaceted role of MLT in regulating gene expression and pathways across plant species under numerous stresses (Table 1). However, further research is needed to explore the tissue-specific effects of MLT at the transcriptional level, which could provide deeper insight into the mechanisms by which MLT enhances stress tolerance. Combining transcriptomic data with other omics tools, such as proteomics and metabolomics (collectively referred to as “panomics”), will provide new insights and enhance our understanding of the protective role of MLT in plant stress adaptation.

### 3.3. Responses at Proteomics Levels

Proteomics is a powerful approach for identifying key proteins and pathways implicated in plant responses to abiotic stresses.^115,116^ Emerging proteomic evidence suggests that exogenous MLT application significantly influences protein expression, post-translational modifications, and signaling networks, thereby enhancing stress tolerance (Table 1). For instance, MLT (500 μM) significantly improved drought tolerance in wheat seedlings by regulating the expression of proteins involved in critical processes such as antioxidant metabolism (MSR, Z-ISO, SFGH, SOD, DHAR, and GME), carbon fixation/metabolism (RuBisCO-3, ENO, and HXK-1), amino acid metabolism (SALH, SP, PHGDH, and AP), and autophagy (RAB5C, RAB11A, and SRPR).^118^ These findings highlight how MLT regulates a network of proteins that promote metabolic adaptations to drought stress.

In cucumber, MLT (1 μM) improved salinity tolerance by upregulating proteins involved in glycolysis, the TCA cycle, and the glyoxylate cycle, along with those regulating ribosome biosynthesis, and lipid and carbohydrate metabolisms.^119^ The modulation of these pathways by MLT suggests its role in maintaining cellular energy homeostasis and supporting metabolic flexibility under salinity conditions. In broccoli, MLT (10 μM) significantly enriched proteins related to sulfur and selenocompound metabolisms, biosynthesis of secondary metabolites, glucosinolates, and peroxisomes, which are crucial for mitigating zinc sulfate (ZnSO_4_) stress.^120^ These proteomic changes demonstrate the influence of MLT on sulfur metabolism and secondary metabolite production, which support its role in detoxification mechanisms under metal stress.

Under waterlogging, MLT (200 μM) triggered proteins associated with carbohydrate transport and metabolism, which activated metabolic processes to enhance stress tolerance to oxygen-deprived conditions in peach^68^. This response emphasizes MLT’s role in regulating energy metabolism to support stress responses under hypoxic conditions. Furthermore, in tomatoes, MLT (100 μM) regulated a wide range of proteins, including HH3.2, NCD2, NFYC1, KELP, PRMT13, HIDM1, HIDM2, CHI3, CB5A, ClpF, and TOC159, which are involved in carbohydrate and energy metabolism, protein translation, amino acid metabolism, and vitamin synthesis^56^. The diversity of protein regulation by MLT in cold-stressed tomato highlights its broad protective role in multiple stress pathways.

In soybean, MLT (10, 50, or 100 μM) regulated the proteins involved in protein synthesis, RNA metabolism, and cell wall biosynthesis, while also enhancing the expression of eukaryotic aspartyl protease family proteins.^119^ This suggests that MLT enhances flooding tolerance by modulating protein networks involved in cellular structure and stress-responsive pathways. Although the data on MLT-mediated proteomic analysis under diverse stress conditions is still limited, we suggest checking Table 1 for additional examples. These studies present the protective role of MLT as a key modulator of proteomic networks involved in stress tolerance. By regulating proteins across a range of metabolic processes (Figure 3), MLT enhances the plant’s ability to cope with diverse abiotic stresses.

### 3.4. Responses at Metabolomics Levels

Metabolomics involves the comprehensive examination of metabolites within a biological system that offer insights into the physiological state of the organism, particularly under stress conditions.^115,121,122^ MLT, a key regulator of various metabolic pathways, plays a significant role in modulating the accumulation of primary and secondary metabolites, thereby enhancing plant stress tolerance. By influencing the biosynthesis and accumulation of multiple compounds, MLT facilitates the adaptation of plants to diverse stresses (Table 1).

In cotton, MLT (50 µmol L^−1^) was shown to regulate the biosynthesis of flavonoids and alkaloids, and accumulation of secondary metabolites, which are crucial for enhancing Cd tolerance.^117^ Likewise, in tomatoes, MLT (100 μM) modulated several metabolic pathways, including tyrosine metabolism and amino acid biosynthesis, which contributed to improved copper tolerance. This regulation of metabolic pathways also influenced the accumulation of flavones, flavonols, and amino acids, which results in copper stress tolerance^71^. A study on apple demonstrated that low concentrations of MLT (0.1 µM) led to the accumulation of key metabolites, such as oxalic acid, L-ascorbic acid, anthocyanins, and lignans, alongside metabolic pathways involved in amino acid and coenzyme biosynthesis (i.e., cyanoamino acid, beta-alanine, alanine, aspartate, and glutamate metabolisms, and pantothenate and CoA), which enhanced nutrient stress tolerance.^123^ These findings highlight MLT’s ability to fine-tune complex metabolic networks, which contribute to enhanced stress tolerance.

MLT also significantly improves drought tolerance by modulating carbon and nitrogen metabolism, as seen in soybeans. In this case, MLT (100 μM) shifted metabolic pathways to enhance the antioxidant defense system and photosynthetic capacity, alongside increased accumulation of amino acids and their derivatives.^124^ Maize plants subjected to drought stress showed a similar accumulation of flavonoid metabolites, including apigenin, luteolin, and quercetin, in response to MLT application (100 μM) to improve drought tolerance^67^. Under salinity stress, MTL (10 μM) promoted the accumulation of amino acids, organic acids, nucleotides, and secondary metabolites in rice, activating different antioxidant pathways and amino acid metabolism to enhance salinity tolerance in rice^62^. Additionally, cotton plants exposed to MLT (50, 100, 200, and 500 μM) showed increased levels of flavonoids, phenolic acids, and secondary metabolites, particularly those involved in glutathione metabolism and linoleic acid biosynthesis, which suggests their role in mitigating salinity-induced oxidative stress^61^. In soybean, MLT (100 μmol L^−1^) exhibited elevated accumulation of major flavonoids and terpenoid metabolites, including uteolin-7-O-(2”-O-rhamnosyl)rutinoside and Hederagenin-3-O-glucuronide-28-O-glucosyl(1,2)glucoside, which were associated with alleviated alkali stress^59^. These studies collectively demonstrate how MLT application leads to the accumulation of diverse metabolites, which support its role in enhanced stress tolerance through regulating various metabolic pathways (Table 1; Figure 3).

### 3.5. Responses at miRNAomics Levels

MicroRNAs (miRNAs) are tiny, non-coding RNAs that control gene expression at the post-transcriptional level and play a critical role in plant responses to abiotic stresses.^125,126^ In recent years, MLT’s impact on miRNAome regulation under abiotic stress has been confirmed across multiple plant species. This offers insights into how it mediates stress responses through epigenetic and post-transcriptional mechanisms. For example, in watermelon, MLT (150 μM) treatment under cold stress led to the downregulation of several miRNAs, including *miR159-5p*, *miR858*, *miR8029-3p*, and *novel-m0048-3p*, which correlated with the upregulation of target genes involved in signal transduction (such as *CDPK*, *BHLH*, *WRKY*, *MYB*, and *DREB*) and detoxification pathways (*LEA* and *MDAR*) that result in enhanced cold tolerance.^127^ Similarly, radish treated with MLT (10–200 μM) exhibited altered miRNA profiles, including *miR157c-3p*, *miR172d-5p*, *miR2111a-3p*, *miRn26*, *miRn32*, *miRn5*, and *miRn33*, which targeted various genes and TFs, significantly improving Cd tolerance^69^.

In Arabidopsis and alfalfa, MLT was found to enhance Cd tolerance by restoring redox balance through the regulation of the *Cu/Zn-SOD* gene by miR398a and miR398b.^128^ These outcomes highlight the vital role of MLT-induced miRNAs in mitigating oxidative stress and heavy metal toxicity. In another study, *miR168a* regulates MLT biosynthesis by targeting the *OMT1* gene in Chinese cabbage under salinity stress. Differences in *miR168a* and *OMT1* expression correlated with higher MLT levels, enhanced antioxidant activity, and better ion homeostasis, which suggests their role in salinity tolerance.^129^ Furthermore, in maize, MLT (10 μM) transformed the expression of several miRNAs, including *zma-miR159a/c*, *zma-miR167a/e/g*, and *zma-miR171d*, which in turn regulated the expression of TFs such as *MYB*, *ARF*, *ERF*, and *bHLH*, and ultimately led to salinity tolerance^81^. These findings highlight the significance of MLT in modulating miRNA expression to enhance plant stress tolerance.

MLT also modulates miRNA expression to regulate plant responses to carbon starvation and drought stress. For instance, in tomatoes, MLT (250 μM) upregulated the expression of *miR171b*, which targets the *GWD* gene, resulting in improved tolerance to carbon starvation by preventing chlorophyll and starch degradation.^130^ In rice, MLT (250 mg L^−1^) influenced the expression of lncRNAs, which then regulated genes involved in pectin and cellulose metabolism, ROS scavenging, and hormone biosynthesis, thereby enhancing Cd tolerance.^131^ Similarly, in cassava, MLT (100 μM) modulated lncRNA expression, linking miRNAs to genes involved in light signaling, fatty acid synthesis, and secondary metabolism, which were crucial for drought stress tolerance.^132^ These studies highlight MLT’s role in miRNA regulation and its potential to mediate complex stress-responsive pathways (Figure 3) and improve tolerance against diverse abiotic stresses through the precise modulation of gene networks.

### 3.6. Responses at Epigenetic/Epigenomics Levels

Epigenetic modifications, particularly DNA methylation, are vital to regulate abiotic stress responses^133,134^. Similar to other omics, MLT’s role in modulating DNA methylation has been reported across various stresses, which results in enhanced stress tolerance by regulating gene expression and metabolic pathways. For instance, in radish, MLT (50 μM) under lead (Pb) stress resulted in demethylation of key antioxidant genes (*RsAPX2*, *RsPOD52*, and *RsGST*) and metal transporter genes/TFs (*RsABCF5*, *RsYSL7*, *RsHMT*, *RsWRKY41*, and *RsMYB2*) promoted ROS scavenging and Pb exclusion, thus contributing to Pb stress tolerance^135^. Similarly, in lettuce, MLT (50, 100, and 200 µM) under salinity stress restored DNA methylation levels, which were reduced by salinity stress, and positively modulated the expression patterns of salinity-related genes, e.g., *SOS1*, *SOS2*, and *HKT1*^136^. MLT’s ability to modulate DNA methylation was further highlighted in bean, where MLT (200 µM) altered DNA methylation and polymorphism patterns that eventually improved the drought and salinity stress tolerance^137^.

Furthermore, MLT also plays a crucial role in postharvest quality maintenance. In peaches, MLT (100 μM) treatment suppressed the expression of *PpPPO* and *PpPOD* while upregulating *PpPAL*, and this phenomenon led to increased phenolic accumulation and reduced browning under chilling stress. This was accompanied by changes in DNA methylation, which were regulated by methylesterase and demethylase activities, thereby enhancing postharvest shelf life^138^. Similarly, in water bamboo shoots, MLT (100 μM) reduced lignin deposition, delayed browning, and preserved phenolic contents under chilling stress by modulating DNA methylation in genes related to the phenylpropanoid pathway^139^. These studies shed light on how MLT plays a dynamic role in regulating DNA methylation to enhance stress tolerance, providing new insights into its potential as an epigenetic modulator to improve plant responses to both abiotic and post-harvest stresses.

### 3.7. Responses at Ionomics Levels

Ionomics, the high-throughput profiling of elemental-ion composition in plants, offers key insights into how plants maintain ionic balance under stress conditions.^140–142^ Recent literature also suggests that MLT application plays a significant role in reprogramming ionomics changes, which contribute to stress tolerance^14,142^, and a few examples are discussed here. In response to nutrient deficiency, MLT (0.1 μM) enhanced overall mineral uptake and relocation in apple plants by adjusting stomatal morphology, boosting antioxidant activities, and activating ion transport genes, as discovered by integrated omics (ionomic, transcriptomic, and metabolomic) analysis^123^. Under nitrate stress, MLT (0.1 mmol L^−1^) stabilized the disrupted balance of macronutrients such as nitrogen (N), phosphorus (P), potassium (K), calcium (Ca), and magnesium (Mg), which promoted optimal nutrient absorption and improved N metabolism in cucumbers^143^. Under drought stress, MLT (100 μM) application enhanced δ^15^N accumulation and upregulated genes related to N uptake and assimilation (e.g., *NRTs* and *AMTs*) in apple plants^144^.

In alkaline-stressed tomato, MLT (0.5 µM) restored ionic balance by reducing sodium (Na^+^) and increasing potassium (K^+^) ion levels in leaves, thus improved the K^+^/Na^+^ ratio, which is essential for cellular homeostasis and stress tolerance^145^. Under salinity stress, foliar MLT (200 µM) in rice significantly inhibited Na^+^ accumulation but improved the uptake and upward transport of K^+^, Ca^2+^, N, and Si, which helped maintain ionic ratios such as K^+^/Na^+^ and Ca^2+^/Na^+^ for salinity tolerance^146^. In maize, MLT (1 µM) under salinity stress enhanced ion balance, particularly by lowering Na^+^ content and improving the K^+^/Na^+^ ratio^147^. Likewise, salinity-stressed tomato seedlings treated with MLT (100 µM) showed similar ionic adjustments with reduced Na^+^ and elevated K^+^, alongside improved nitrate assimilation^148^. In sorghum, MLT (100–200 µM) enhanced salinity tolerance by conserving ionic balance, particularly Na^+^, K^+^, and Ca^2+^ homeostasis, and thus strengthened the cell membrane stability and antioxidant defense systems^149^.

Under heavy metal stress, MLT also conferred ionomic protection. In nickel (Ni)-stressed tomato, MLT (100 µM) reduced Ni accumulation and improved nutrient uptake through better root architecture and ion homeostasis^150^. In response to vanadium (V) stress, MLT (5 μmol L^−1^) reduced V translocation from root to shoot and promoted mineral nutrient accumulation across pepper genotypes^151^. Altogether, these outcomes feature the crucial role of MLT in regulating ion uptake, transport, and compartmentalization across stress types. By stabilizing ionic ratios (e.g., K^+^/Na^+^ and Ca^2+^/Na^+^), enhancing nutrient acquisition, and mitigating the accumulation of toxic ions, MLT-driven ionomic responses emerge as a vital mechanism underlying stress tolerance and crop improvement.

### 3.8. Responses at Phenotypic Levels

Phenotypic or phenomic traits provide a direct measure of plant growth, development, and structural adaptations under stress conditions^83^. MLT plays a crucial role in regulating these traits, enhancing abiotic stress tolerance by sustaining biomass, promoting root development, and mitigating stress-induced morphological damage. Several studies have investigated the effects of MLT on phenotypic traits under various stress conditions (a few examples are discussed below), highlighting its potential to enhance plant growth and yield stability.

MLT treatment has shown varying effects on plant morphology, depending on the type of stress and the plant species. Under heat stress, MLT (50–200 µmol L^−1^) maintained plant height and increased stem diameter, particularly at 50 µmol L^−1^, indicating its protective role in maintaining structural integrity in potato seedlings^52^. Similarly, in tomato under Cd stress, MLT (100 µmol L^−1^) improved biomass accumulation and leaf size, although its effect was diminished in *COMT-PCS* co-silenced plants, which suggests a partial dependency on *PCS*-mediated pathways^152^. In contrast, MLT (10 μM) treatment in lettuce under salinity stress did not significantly improve leaf area or biomass, though long-term MLT (150 μM) application (6–10 weeks) has been found to positively impact plant growth in other species like sorghum and lettuce^83,153,154^. These findings suggest that the efficiency of MLT in enlightening stress-induced phenotypic alterations may vary depending on species, stress duration, and concentration.

Root traits are particularly responsive to MLT under stress conditions. In melon seedlings exposed to copper stress, MLT (100 µmol L^−1^) significantly improved root length, surface area, and volume compared to untreated stressed plants, which feature its unique role in improving root system architecture^89^. Likewise, in cotton, MLT (100 µmol L^−1^) mitigated drought-induced reductions in root growth, increasing root surface area, volume, and specific root length, while also delaying root senescence^155^. In another cotton study, MLT (10 μM) seed priming improved drought tolerance in the sensitive variety “L-799” by enhancing plant height, root length, and leaf area. At the same time, its effects were limited in the drought-tolerant variety “Suraj,” except for an increase in leaf area^156^. Similar findings were observed in rapeseed, where MLT (100 µM) enhanced lateral and taproot length under drought stress in certain genotypes but had no significant impact on biomass^157^. However, in peach seedlings under waterlogging, MLT (200 µM) had no effect on root biomass but protected root structure, which suggests a role in maintaining root integrity rather than promoting growth^68^. In rice, MLT (10 µM) improved survival rates under salinity stress, but no phenotypic differences were observed under non-stressed conditions, indicating its specific action under stress environments^62^.

MLT has also been shown to improve reproductive traits under stress conditions. In rice exposed to arsenic, MLT enhanced root and shoot growth, increased grain number and grain weight, and reduced arsenic bioaccumulation, particularly in the more stress-sensitive genotype KT^90^. Similarly, in soybean, MLT (100 µM) improved plant growth and yield by increasing pod formation, grain number, and 100-grain weight under drought stress^124^. These findings highlight MLT’s role in safeguarding yield-related traits, which are critical for agricultural productivity under stress conditions. However, MLT’s effects are highly species- and stress-dependent, which demands further research to optimize dosage, application timing, and mode of action.

### 3.9. Responses at Microbiomics Levels

Plant microbiome plays a crucial role in modulating stress tolerance by influencing nutrient uptake, phytohormone signaling, and systemic resistance mechanisms^158^. Despite extensive work on MLT-enabled stress responses, yet little is known about its interaction with the plant-associated microbiome under abiotic stress conditions. Thus, this corresponds to a critical research gap, as MLT may serve as a key regulator of plant–microbe interactions, which potentially can enhance microbial diversity and functional stability under stress.

Recent studies suggest that MLT applications can induce substantial changes in rhizospheric microbial communities, thereby boosting tolerance to various abiotic stresses. For instance, it has been exhibited that MLT application (1 mmol L^−1^) in barley under cold stress enhanced photosynthetic carbon assimilation and redox homeostasis through modifications in microbial diversity. Notably, MLT altered the composition of nitrogen-cycling microorganisms, which results in influenced amino acid synthesis and metabolism in the rhizosphere for enhanced cold tolerance^159^. Similarly, another study found that MLT (2 mmol L^−1^) under drought stress in barley enhanced non-structural carbohydrate metabolism and antioxidant enzyme activity. Metagenomic analysis discovered that MLT increased the abundance of microbes involved in carbohydrate and carboxylate degradation, while reducing fatty acid and lipid degradation pathways and advising its role in improving nutrient cycling under drought stress^160^.

Under waterlogging stress, three studies in apple presented critical insights into MLT’s role in modifying microbial communities to improve stress tolerance^161–163^. These studies reported that MLT (400 µM) improved nitrogen (N) utilization by upregulating genes related to N transport and metabolism in apple plants, whereas also recruiting beneficial microbes such as *Azoarcus*, *Pseudomonas*, *Nocardioides*, and *Hydrogenophaga*. These microbes showed positive correlations with soil nutrient levels and plant growth. Moreover, network and metabolomic analysis indicated that MLT and dopamine co-regulated the endophyte community, promoting the synthesis of flavonoids, coumarins, and organic acids that contributed to waterlogging tolerance. Overall, these outcomes highlight a key function of MLT in shaping the plant microbiome to enhance nitrogen acquisition and physiological adaptation to stressful conditions^161–163^.

Generally, MLT contributes to stress tolerance; however, its interaction with fertilizers and rhizosphere microbial communities can generate complex and sometimes astonishing responses. For example, a previous study examined the combined effects of foliar-applied MLT (100 μmol L^−1^) and urea on soybean productivity and rhizosphere microbial composition. Urea application alone significantly increased yield; the combined treatment of MLT and urea unexpectedly reduced productivity. Transcriptomic analysis showed that MLT altered urea-responsive gene expression patterns, redirecting plant metabolic activity toward stress-related signaling pathways rather than growth-promoting processes. Moreover, microbiome profiling showed that although urea and MLT individually reshaped microbial abundance, their combined application largely neutralized these effects^164^. These findings emphasize the need for careful optimization of MLT use in conjunction with conventional fertilizers to prevent unintended antagonistic interactions that could compromise crop performance.

In addition to stress tolerance, a study investigated the synergistic effects of MLT (50 µM) and *Pseudomonas putida* in improving thiamethoxam-induced toxicity in *Brassica juncea*. Their findings showed that combined treatment significantly upregulated organic acid accumulation (e.g., malic, citric, and fumaric acids), which helped stress mitigation and energy generation^165^. Additionally, it has also been determined that MLT (100 μmol L^−1^) promoted N uptake and microbial diversity under drought in tea Crabapple (*Malus hupehensis*), which indicates a dual role in nutrient assimilation and microbial recruitment^166^. A recent study in *Diospyros lotus* demonstrated that MLT (100 μM) enhances drought tolerance and recovery by reducing oxidative stress, modulating sugar metabolism and flavonoid biosynthesis, and recruiting beneficial microorganisms (e.g., *Skermanella*, *Nocardioides*, *Ralstonia*, and *Rhodococcus*). Metabolome analysis discovered that 3-hydroxy-butanoic acid, significantly induced by MLT, may enable microbial recruitment, signifying a novel mechanism by which MLT shapes the microbiome to improve drought tolerance^167^.

Another recent study highlighted the potential of MLT-producing plant growth-promoting bacteria (PGPB) in stress management. A novel strain of *Bacillus safensis* “EH143” was identified as a halotolerant and heavy metal-resistant rhizobacterium capable of synthesizing MLT through the tryptophan-tryptamine-serotonin-MLT pathway. This bacterium significantly enhanced phosphorus/calcium uptake and the K^+^/Na^+^ ratio under salinity stress, and reduced Cd accumulation in soybean plants^168^.

Based on the above arguments, we conclude that MLT is a critical modulator of plant–microbe interactions and plays a key role in shifting the rhizosphere and endophytic microbial communities to enhance plant stress tolerance. The observed effects are stress-specific, with MLT enabling N uptake, promoting microbial diversity, and regulating oxidative stress in various environments. However, the antagonistic effects of MLT with N fertilizers highlight the need for optimized application strategies.

Therefore, future research should focus on harnessing the precise molecular mechanisms underlying MLT-enabled microbial shifts and stress responses. Metagenomic and transcriptomic approaches can help identify key microbial taxa and metabolic pathways influenced by MLT under stress conditions. Furthermore, the examination of MLT-producing microbes in biofertilizer development could provide sustainable solutions for crop improvement under climate change conditions.

## 4. Melatonin-Enabled Omics Responses Under Combined Abiotic Stresses: A Relatively Less explored Area

**Figure 4.**
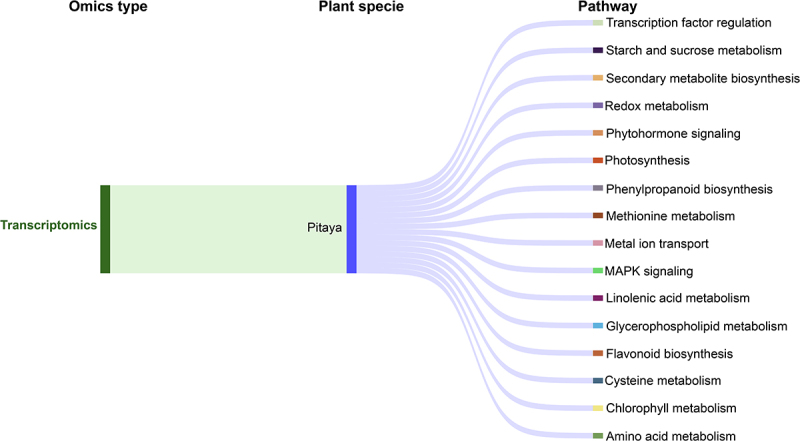
A Sankey diagram illustrates the connection between melatonin-enabled transcriptome responses in pitaya and stress-responsive pathways under combined abiotic stress conditions. Data was obtained from all the studies cited in Section 4.

Abiotic stresses rarely occur in isolation in natural and agricultural environments; instead, plants often face combined abiotic stresses together. However, understanding the unique mechanisms underlying MLT-enabled stress tolerance entails an integrated multi-omics (i.e., panomics) approach rather than a single-stress analysis. Though single-stress studies deliver valuable insights, but they often overlook the complex interactions between stress combinations (*n*≥2). In this context, panomics-driven investigations can bridge this gap by identifying key regulatory hubs and stress-responsive pathways that are unique to combined stress conditions (Figure 4), which can ultimately guide more effective stress-smart crop improvement.

At the genomic level, MLT application has been shown to modulate the expression of key gene families associated with stress tolerance. For instance, in pitaya, MLT (0.099 mM L^−1^) upregulated the expression of several *HuSBPs* genes under Cd + salinity + MLT, Cd + drought + MLT, and Cd + drought + salinity + MLT combinations, which highlighted its role in heavy metal detoxification and osmotic stress adaptation^111^. Similarly, under a combined nickel + drought stress, MLT (100 µM) enhanced the expression of *HuMADS-box* genes, which are known regulators of stress-responsive transcriptional networks^110^. Moreover, in response to a different stress combination (drought + heat + vanadium), MLT (100 µM) modulated the expression of several *HuAPXs*^109^ and *HuHMAs*^108^ genes in pitaya, which are crucial for oxidative stress mitigation and metal ion transport. These findings imply that MLT not only improves individual stress tolerance but also rewrites transcriptional reprogramming under combined abiotic stresses. Therefore, future work should focus on the genetic manipulation of these MLT-responsive genes to develop stress-smart crops.

At the transcriptomic level, MLT plays a vital role in shaping gene expression patterns to counteract the negative effects of combined abiotic stresses. For example, transcriptome profiling of pitaya under heat + drought + vanadium combination with MLT (100 μM L^−1^) highlighted 70 conserved differentially expressed genes involved in transcription regulation, oxidative stress responses, metal ion homeostasis, lipid biosynthesis, and phytohormone signaling. Notably, five novel genes (*HU09G00081*, *HU09G01329*, *novel.3095*, *novel.2606*, and *novel.1274*) were specifically upregulated in response to combined stress with MLT treatment, and features their importance in stress adaptation mechanisms^169^.

A comparative transcriptome analysis of pitaya under different stress combinations, including (1) Cd + salinity + drought vs. Cd + drought + salinity + MLT, (2) Cd + drought vs. Cd + drought + MLT, and (3) Cd + salinity vs. Cd + salinity + MLT, discovered 141 common differentially expressed genes associated with key pathways such as phytohormone signaling, photosynthetic antenna proteins, and alpha-linolenic acid metabolism^170^. Additionally, MLT-induced transcriptomic shifts under Cd + salinity + MLT conditions activated secondary metabolite biosynthesis, porphyrin and chlorophyll metabolism, and glycerophospholipid pathways, further enhancing stress tolerance. Similarly, Cd + drought + MLT treatments enriched pathways related to flavonoid biosynthesis, MAPK signaling, phenylpropanoid biosynthesis, and amino sugar metabolism, which sheds light on the vital role of MLT in adjusting stress-adaptive metabolic networks.^170^

Further supporting these findings, another transcriptomic study in pitaya identified 13 common differentially expressed genes across multiple stress combinations: (1) salinity vs. salinity + MLT, (2) copper vs. copper + MLT, and (3) salinity + copper vs. salinity + copper + MLT^171^. These genes were linked to key pathways, including carbon fixation, starch and sucrose metabolism, glycolysis/gluconeogenesis, and flavonoid biosynthesis in salinity vs. salinity + MLT treatments. In contrast, copper vs. copper + MLT treatments showed enrichment in phenylpropanoid biosynthesis, cysteine and methionine metabolism, and alpha-linolenic acid metabolism. On the other hand, salinity + copper vs. salinity + copper + MLT treatments influenced photosynthetic proteins, glucuronate interconversions, and MAPK signaling. These extensive outcomes highlight how MLT dynamically reconfigures transcriptomic responses in a stress-specific manner and fine-tunes the plant defense mechanisms against combined stresses^171^.

These studies support the idea that MLT does not act through a single pathway but rather regulates a complex network of transcriptional and metabolic reprogramming to confer combined stress tolerance (Figure 4). To visualize this complexity, Figure 4 illustrates MLT-modulated pathways specifically under combined abiotic stress conditions in pitaya. This integrated view highlights how MLT targets overlapping yet stress-specific pathways such as phytohormone signaling, antioxidant responses, and ion homeostasis, depending on stress context and plant genotype. By advancing panomics studies and applying these insights to crop breeding programs, we can harness MLT’s full potential for managing combined abiotic stresses across plant species.

## 5. Crosstalk Between Omics Levels: Integrated Data for Comprehensive Insights to Empower Melatonin-Enabled, Stress-Smart Agriculture

Understanding complex stress responses requires a complete approach that moves beyond single-layer analysis. In this context, panomics analysis provides a comprehensive perspective on stress adaptation mechanisms. Whereas single-omics layer offers valuable insights, yet they often fail to capture the complex regulatory networks underlying stress tolerance^172–174^. By integrating multi-omics/panomics layers, we can resolve the crosstalk between stress-related pathways, identify novel regulatory targets, and develop more precise strategies for MLT-enabled, stress-smart agriculture.

Recent studies highlight the power of integrated omics in elucidating the MLT magic in stress mitigation. For instance, MLT-enabled integrated transcriptomic, miRNAome, and metabolomic analysis of maize reported that MLT (10 μM) enhances salinity tolerance in germinating seeds by regulating antioxidant activity, phytohormone signaling (cytokinin and auxin), and metabolic adaptation. miRNA-seq analysis highlighted MLT-induced differential expression of miRNAs targeting different TFs. In contrast, metabolomic profiling showed increased secondary metabolites, nucleotides, and vitamins, which strengthen the power of MLT in stress adaptation^81^. Another integrated transcriptome and metabolome analysis of rice identified key gene-metabolite networks involved in linoleic acid and amino acid metabolisms, which contribute to MLT (10 μM)-mediated salinity tolerance^62^. A separate integrated study showed that MLT (5 μM) enhances seed germination under salinity by upregulating antioxidative pathways and modulating phytohormone balance. Transcriptomic analysis identified stress-responsive genes associated with antioxidative activities and hormone signaling, whereas metabolome profiling noticed increased levels of non-enzymatic antioxidant organic acids and amino acids^79^. Similarly, in wheat, integrated transcriptomic and metabolomic analysis highlighted MLT (10 mM)-induced activation of tryptophan metabolism and flavonoid biosynthesis under drought stress. Key metabolites, including tryptamine and 6-hydroxymelatonin, were altered, and stress-responsive TFs (AP2/ERF, WRKY, bZIP, and MYB) were differentially regulated, which together illustrate the role of MLT in metabolic and transcriptional reprogramming^88^.

In cotton, transcriptome and metabolome integration showed that MLT (50 and 100 µmol L^−1^) application modulates valine, leucine, and isoleucine degradation, ABC transporter pathways, and alpha-linolenic acid metabolism to mitigate Cd toxicity^117^. Similarly, in tomatoes, MLT (100 μM)-enabled transcriptomic and metabolomic changes activated phytohormone signal transduction, phenylpropanoid biosynthesis, and glutathione metabolism to enhance copper stress tolerance.^71^

The integration of transcriptomics with other omics layers, such as proteomics and ionomics, has also delivered new insights into stress tolerance. For instance, in waterlogged peach seedlings, integrated transcriptomic and proteomic analysis observed that MLT (200 μM) priming regulates key TFs (AP2/ERF, HSF, and WRKY) and enhances glycolysis/gluconeogenesis pathways. In contrast, their correlation analysis identified *ERF071*, *ADH*, and *PCO* genes as promising targets for waterlogging tolerance^68^. Additionally, integrated physiological, transcriptomic, and proteomic investigations in wheat discovered that MLT (100 μM) enhances drought tolerance by increasing jasmonic acid (JA) levels, upregulating JA-related genes (*LOX1.5* and *LOX2.1*), TFs (*HY5* and *MYB86*), and genes involved in lignin biosynthesis and starch/sucrose metabolism. MLT also mitigated drought-induced oxidative damage, which features its potential as a biostimulant^76^. In tomatoes, integrated transcriptomic and proteomic analysis found that the MLT (100 μM) treatment resulted in the upregulation of genes and proteins related to TFs, signal transduction, environmental adaptation, and chloroplast integrity maintenance under cold stress^56^. Furthermore, in apple plants, an integrated ionomic, transcriptomic, and metabolomic investigation reported that MLT (0.1 µM) enhances nutrient absorption and stress tolerance by upregulating glutathione metabolism, metal ion transport genes, and key metabolites such as oxalic acid, L-ascorbic acid, anthocyanins, lignans, and melatonin^123^.

**Figure 5.**
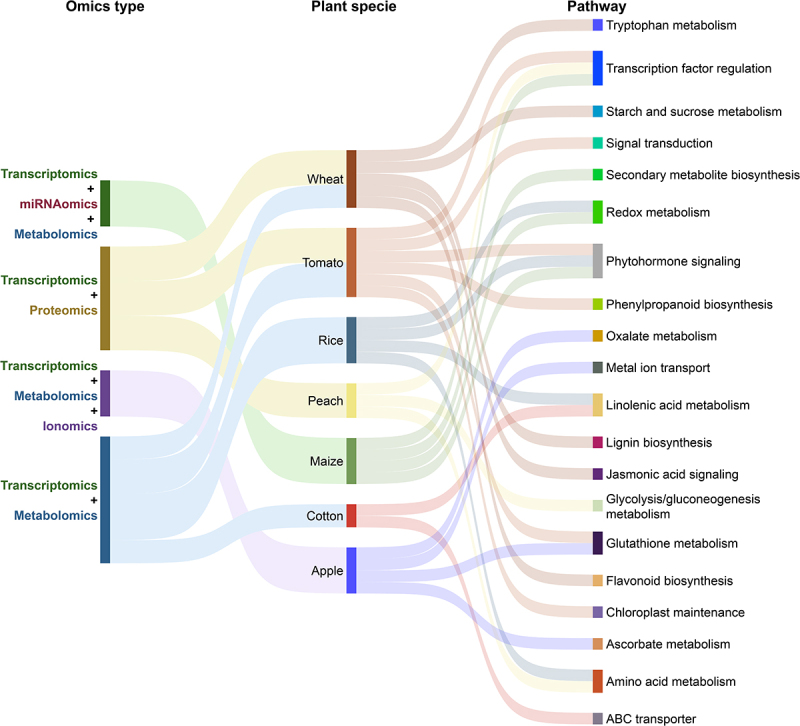
A Sankey diagram illustrates the convergence of multiple omics layers with different plant species and melatonin-regulated biological pathways under integrated multi-omics studies and different stress conditions. The figure highlights the crosstalk between omics layers and emphasizes the systemic influence of melatonin on complex regulatory networks associated with different abiotic stress tolerance. Data was obtained from all the studies cited in Section 5.

These integrated panomics studies advocate that MLT modulates diverse molecular pathways in a stress-specific manner across plant species (Figure 5). As featured in Figure 5, this depiction helps identify conserved regulatory nodes and potential targets for stress-smart breeding strategies. In this context, future research should examine the integration of single-cell omics and spatial transcriptomics to explore tissue-specific responses to MLT under stress conditions. Moreover, expanding panomics studies across diverse crop species and field conditions will provide deeper insights into the real-life applicability of MLT in stress-smart agriculture.

## 6. What Do We Need to Do with the Newly Identified Targets from Omics? A Genetic Engineering Perspective

**Figure 6.**
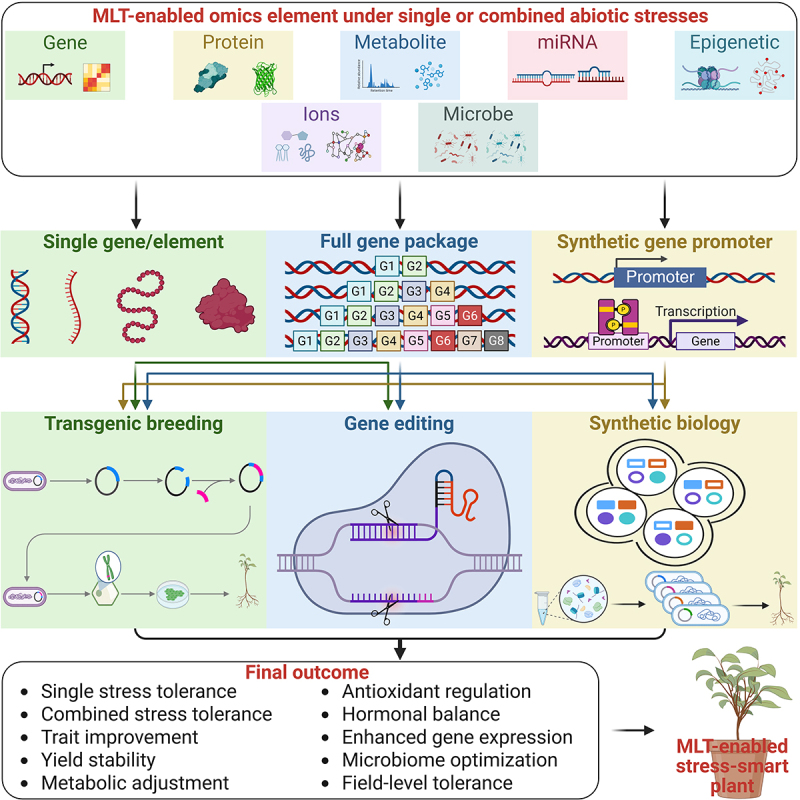
Strategic utilization of melatonin (MLT)-enabled omics elements to engineer stress-smart plants through transgenic breeding, gene editing, and synthetic biology. Melatonin-regulated omics elements can be translated into actionable targets via single-gene approaches, multi-gene stacking, or synthetic regulatory designs. The integration of such approaches leads to the design of MLT-enabled, stress-smart plants. Created with BioRender.com.

With the blast of data (molecular elements) from MLT-enabled omics studies, we now stand at a critical stage: what next? A conceptual context demonstrating how these elements can be functionally applied through various engineering approaches is shown in Figure 6. As discussed in earlier sections and Table 1, diverse MLT-responsive genes, proteins, metabolites, miRNAs, and microbial regulators have been identified under both single and combined abiotic stress conditions across plant species. However, discovery is only the first step; the larger challenge lies in how we utilize these targets to design climate-smart crop plants.

One key question is: how many of these MLT-enabled elements are truly actionable? Can we engineer individual genes for broad-spectrum stress tolerance, or do we require multi-gene strategies (i.e., full gene package) to address complex stress combinations, as argued in^5^? The current evidence proposes that single-gene manipulations may be effective under control or single stress conditions (Table 2), but combined abiotic stresses often involve multiple, overlapping, and dynamic signaling pathways. This calls for “gene stacking” or “network-based engineering” approaches to simultaneously modulate stress signaling, antioxidant defenses, metabolic fluxes, and phytohormone balance (Figure 6).^5^ The development of precision tools such as CRISPR/Cas9 has made it feasible to edit or fine-tune these stress-responsive elements. For instance, overexpression/knockout of MLT biosynthesis-related genes has been shown to enhance multiple stress tolerance (see Table 2 for some examples). These studies clearly emphasize the power of targeted genetic engineering guided by omics data.

**Table 2.** Examples of genetic engineering of melatonin‑associated genes to improve multiple abiotic stress tolerance across plant species.

Stress type	Plant specie	Gene	Engineering type	Key stress tolerance mechanism	Reference
Salinity	Tomato (*Solanum lycopersicum*)	*SlCOMT1*	OE	Increased endogenous MLT accumulation under salinity Reduced ROS and increased proline content *SlCOMT1* expression positively correlated with MLT levels Recovered physiological responses under high salinity	175
Salinity	Tomato (*Solanum lycopersicum*)	*SlCOMT1*	OE	Activated SOS pathway to maintain Na^+^/K^+^ homeostasis Enhanced antioxidant enzyme activities and AsA/GSH levels Maintained nutrient homeostasis Upregulated key stress-related genes (*AREB1*, *MAPK1*, *WRKY33*, etc.)	176
Salinity	Arabidopsis	*VvSNAT1*	OE	Increased MLT content and improved salinity stress responses Reduced H_2_O_2_ and MDA accumulation (less oxidative damage) Promoted growth such as germination, root length, and fresh weight Protected chloroplast-localized SNAT enhances stress tolerance	177
Salinity and osmotic	Tobacco (*Nicotiana benthamiana*)	*VvASMT1*	OE	Enhanced MLT production and antioxidant enzyme activity Increased proline and MDA levels under stress Improved root growth and water retention (RWC) Reduced ROS accumulation and leaf wilting	178
Alkaline	Apple (*Malus domestica*)	*HIOMT*	OE	Elevated endogenous MLT and organic acid levels Improved root growth and photosynthetic efficiency Enhanced antioxidant activity and membrane stability Maintained ionic balance	179
Nitrogen deficiency	Apple (*Malus domestica*)	*MdASMT9*	OE	Improved light harvesting and photosynthetic heat transfer Enhanced TCA cycle and amino acid metabolism Upregulated *MdHY5* expression, boosting N transporter genes Promoted N uptake via *MdNRT2.1*/*2.4* activation	180
Mercury	Arabidopsis	*LaCOMT*	OE	Conferred mercury tolerance by ROS scavenging Boosted antioxidant enzyme activities Increased cellular antioxidant accumulation Modulated ROS homeostasis under mercury toxicity	181
Heat	Apple (*Malus domestica*)	*MdASMT9*	OE	Reduced ABA via suppression of *MdNCED1*/*3* by *MdWRKY33* Promoted stomatal opening for cooling Protected chloroplasts and enhanced antioxidant defenses Increased autophagy via *MdATG18a* activation	182
Heat	Rose (*Rosa hybrida*)	*RhCOMT1*	OE and VIGS	*RhCOMT1* translocation to chloroplasts under heat Enhanced Chl content and reduced ion leakage Improved tolerance via ROS modulation *RhCOMT1* silencing reduced stress tolerance	183
Cold	Watermelon (*Citrullus lanatus*)	*ClCOMT1*	OE and knockout	Activated CBF pathway via NO and H_2_S signaling Upregulated expression of *ClNR1* and *ClLCD* genes NO-H_2_S positive feedback loop drives cold tolerance SNP/NaHS restored cold response in *ClCOMT1* knockout	184
Drought	Tobacco (*Nicotiana benthamiana*)	*CrCOMT*	OE	Increased proline, antioxidant enzyme activity, and MLT levels Lower MDA and electrolyte leakage, and higher membrane stability Improved photochemical efficiency and survival under stress Upregulated expression of drought- and ROS-related genes	185
Drought	Poplar (*Populus tomentosa*)	*PtoASMT*	OE	Reduced ROS under drought via enhanced MLT biosynthesis Altered expression of *PR* and *JAZ10* genes Knockout plants were ROS-sensitive and stress-susceptible	186
Drought	Orange (*Poncirus trifoliata*)	*PtCOMT5*	OE and knockdown	Promoted root development and MLT accumulation *PtCOMT5* activated by *PtbHLH28* and *PtABF4* genes Enhanced drought tolerance via ABA-signaling module CRISPR knockout showed reduced tolerance phenotype	187
Light	Arabidopsis	*MePMTR1*	OE	Improved photosynthesis and biomass Delayed dark-induced senescence Lower ROS accumulation and better Chl retention Highlighted receptor-mediated MLT signaling in growth	188

Abbreviations: abscisic acid (ABA); ascorbic acid (AsA); chlorophyll (Chl); glutathione (GSH); hydrogen sulfide (H_2_S); hydrogen peroxide (H_2_O_2_); malondialdehyde (MDA); melatonin (MLT); nitric oxide (NO); nitrogen (N); overexpression (OE); reactive oxygen species (ROS); relative water content (RWC); salt overly sensitive (SOS).

Moreover, “plant synthetic biology” is now rapidly emerging as a powerful companion to classical genetic engineering^189–191^. By designing synthetic promoters responsive to MLT or engineering synthetic gene circuits that modulate antioxidant or hormone pathways in a stress-inducible manner, we can go beyond natural gene regulation (Figure 4)^192^. For instance, incorporating MLT-responsive *cis*-regulatory elements into synthetic constructs could enable spatiotemporal control over gene expression. Synthetic pathways for MLT biosynthesis could also be introduced into crops with inherently low endogenous MLT levels, resulting in enhanced inherent tolerance without the need for repeated exogenous application. A recent study confirmed this approach by engineering multiple BUFFER-based synthetic genetic circuits in two major crops (i.e., soybean and cotton), which led to a 31-fold upsurge in endogenous MLT levels and enhanced salinity tolerance in soybean seeds and pathogen resistance (*Verticillium dahlia*) in cotton, without a yield penalty^193^. This clearly features the potential of synthetic biology in not only boosting nutritional value but also improving stress tolerance through MLT-enabled pathway engineering.

Regardless of these progresses, many important questions remain, including (1) How many of the MLT-activated pathways are redundant, and how many are essential? (2) Can a core set of MLT-regulated genes be identified for universal stress tolerance, or are these responses highly species- and stress-specific? (3) Can engineering the microbiome or epigenetic machinery propose complementary routes to enhance or stabilize MLT responses in variable field conditions? Addressing these critical questions will require a systems-level approach, integrating data from multi-omics, network biology, and functional genomics. Importantly, future efforts should also emphasize field validation, as many omics-driven targets show promise in controlled settings but remain unverified in natural field agricultural environments.

## 7. Conclusion, Research Gaps, and Future Perspectives

MLT has emerged as a “master regulator” of plant stress responses, influencing a diverse array of genes, proteins, metabolites, and regulatory elements involved in abiotic stress tolerance (Table 1). Through multi-omics investigations, recent studies have discovered how MLT reprograms stress-responsive pathways across plant species and stress types. As illustrated via Sankey plots (Figures 3, 4 , and 5), a growing body of data now identifies common and unique pathways, such as phytohormone signaling, redox regulation, photosynthesis, flavonoid biosynthesis, MAPK cascades, carbohydrate, amino acid, and secondary metabolism, etc., that respond to single (Figure 3) and combined abiotic stresses (Figure 4), and are shaped by integrated multi-omics layers (Figure 5). These pathway-level insights highlight key regulatory nodes and omics-responsive elements (e.g., transcripts, proteins, or metabolites) that serve as strategic targets for engineering stress tolerance. To translate these findings, it is imperative to move toward targeted manipulation of MLT-responsive omics elements, whether through transgenic breeding, multi-gene stacking, or synthetic biology approaches, such as the use of stress- or tissue-specific synthetic promoters (see Figure 6 for a schematic workflow). Harnessing these tools will enable the development of MLT-enabled stress-smart crops designed for future climates. Notably, such insights are especially crucial under combined stress conditions, where the complexity of plant responses demands a holistic view of molecular interactions and regulatory feedback.

Despite notable progress, several knowledge gaps persist. Most current studies are focused on controlled conditions and single stress states, which limit our understanding of tissue-specific and field-relevant responses to MLT under combined stresses. The epigenetic foundations of MLT-regulated stress tolerance, including DNA methylation and histone modifications, remain largely unknown. Moreover, the integration of proteomics, ionomics, and metabolomics with transcriptomic data is crucial for discovering post-transcriptional and post-translational mechanisms that fine-tune stress responses. To improve applicability, future work should embrace high-throughput phenotyping, AI/ML-assisted data integration, and functional validation to bridge the gap between lab-based omics discoveries and practical field deployment.

To fully leverage the wealth of omics-driven knowledge, we must transition from descriptive biology to actionable genetic and synthetic engineering. This includes adopting CRISPR/Cas-based genome editing to fine-tune MLT-responsive networks (e.g., “full gene package,” engineering synthetic gene circuits, and leveraging microbiome or epigenome editing) to alleviate stress tolerance. Ultimately, a synergistic combination of MLT biology, multi-omics integration, synthetic biology, and precision breeding can alter our ability to design climate-smart, high-performing crops.

In brief, MLT-responsive omics data present a rich platform for next-generation crop improvement. What is now needed is a translational pipeline to convert these multi-omics discoveries into substantial, field-ready solutions for sustainable and climate-smart agriculture.
